# MiR-30a-5p Inhibits Epithelial-to-Mesenchymal Transition and Upregulates Expression of Tight Junction Protein Claudin-5 in Human Upper Tract Urothelial Carcinoma Cells

**DOI:** 10.3390/ijms18081826

**Published:** 2017-08-22

**Authors:** Yueh-Hua Chung, Sung-Chou Li, Ying-Hsien Kao, Hao-Lun Luo, Yuan-Tso Cheng, Pey-Ru Lin, Ming-Hong Tai, Po-Hui Chiang

**Affiliations:** 1 Institute of Biomedical Sciences, National Sun Yat-sen University, Kaohsiung 80424, Taiwan; mc6912@gmail.com; 2 Genomics and Proteomics Core Laboratory, Department of Medical Research, Kaohsiung Chang Gung Memorial Hospital and Chang Gung University College of Medicine, Kaohsiung 83301, Taiwan; raymond.pinus@gmail.com; 3 Department of Medical Research, E-Da Hospital, Kaohsiung 82445, Taiwan; ed105156@edah.org.tw; 4 Department of Urology, Kaohsiung Chang Gung Memorial Hospital and Chang Gung University College of Medicine, Kaohsiung 83301, Taiwan; alesy1980@gmail.com (H.-L.L.); ytsocheng@gmail.com (Y.-T.C.); 5 Division of Hepato-Gastroenterology, Kaohsiung Chang Gung Memorial Hospital, Kaohsiung 83301, Taiwan; lin.pr3@gmail.com

**Keywords:** epithelial-to-mesenchymal transition, microRNA, tight junction, transcriptome, upper tract urothelial carcinoma

## Abstract

The involvement of microRNAs (miRNAs) in cancer development and their potential as prognostic biomarkers are becoming increasingly known. However, the signature of miRNAs and their regulatory roles in tumorigenesis of upper tract urothelial carcinoma (UTUC) remain to be elucidated. This study aimed to profile the miRNA expression pattern in UTUC tumor tissues and identify candidate miRNAs with prognostic and/or therapeutic functions. Methods and Results: We collected 22 UTUC tissue and adjacent normal tissues samples from patients who underwent nephroureterectomy. The miRNAs signatures of three selected UTUC samples using next-generation sequencing showed that miR-30a-5p was significantly downregulated in UTUC tumors compared to adjacent normal tissues. The differentially-expressed miRNAs were specifically validated by quantitative real-time polymerase chain reaction. In addition, the miRNA expression signatures were analyzed with the transcriptome profile characterized by microarray. Further in vitro studies indicated that overexpression of miR-30a-5p significantly suppressed proliferation, migration, and epithelial-to-mesenchymal transition (EMT) in cultured BFTC-909 UTUC cells. As a potential target gene of miR-30a-5p in the tight junction pathway suggested by the pathway enrichment analysis, the reduced expression of tight junction protein claudin-5 in UTUC cells was demonstrated to be upregulated by miR-30a-5p genetic delivery. Conclusions: Taken together, our findings demonstrated that miR-30a-5p inhibits proliferation, metastasis, and EMT, and upregulates the expression of tight junction claudin-5 in UTUC cells. Thus, miR-30a-5p may provide a promising therapeutic strategy for UTUC treatment.

## 1. Introduction

Urothelial carcinoma (UC) is the most common cancer of the epithelium lining the urinary tract, and can be divided into upper urinary tract cancers, including renal pelvis carcinoma and ureteral carcinoma, as well as lower urinary tract cancers, including bladder cancer and urinary tract cancer (urethra cancer). UC is a malignant tumor that has become the fourth and eighth most common cancer in men and women, respectively, in the United States [1]. Upper tract urothelial carcinoma (UTUC) is a relatively rare condition in urothelial tumors in Western countries. However, there is specific high incidence in Taiwan [1]. Therefore, developing new therapeutic and diagnostic strategies for the treatment is necessary.

MicroRNAs (miRNAs) are short (19–22 nucleotides), non-coding RNAs that silence gene expression at a post-transcriptional level via suppression of sequence-complementary mRNA targets [2]. It is already known that miRNAs are involved in diverse biological processes, including cell differentiation, proliferation, and apoptosis [3]. Numerous miRNAs have been shown to display tumor suppressor activity, while others reportedly act as oncogenes [4,5]. The expression levels of these RNAs are altered in many human tumors, resulting in distinct miRNA networks in various tumor types, and have been implicated in several cancers [6,7,8,9]. Recent studies have shown that miRNA expression may be used as prognostic markers to identify UTUC in formalin-fixed paraffin-embedded tissues or serum samples [10,11,12]. Thus, analysis of the miRNA profile may provide insights into the molecular mechanisms involved in UTUC tumorigenesis and therapeutics.

Previous studies have shown that miR-30a-5p was downregulated in cancers, and acts as a tumor suppressor that suppresses cancer cell proliferation, migration, and invasion [13,14]. Moreover, miR-30a-5p has been reported as a biomarker in patients with focal and segmental glomerulosclerosis [15], and modulates epithelial-to-mesenchymal transition (EMT) in colorectal, pulmonary, and gastric cancers [14,16,17]. As EMT plays a critical role in the increased migration and invasion of cancer cells, recent studies have revealed that miR-30a inhibits the migration and invasion of breast cancer cells via suppressing EMT progression [18,19]. To date, the molecular mechanisms of miR-30a-5p in the EMT of UTUC cells remain unclear and need further investigation. Moreover, little is known about whether miRNAs are differentially expressed between UTUC and adjacent normal tissues, or the precise mechanism by which the aberrantly-expressed miRNAs contribute to tumor growth and the spread of UTUC cells.

In this study, we first used next-generation sequencing (NGS) and microarray techniques to profile the miRNA expression and their putative mRNA targets in UTUC tissues, and identified a unique miRNA signature, the downregulation of miR-30a-5p in UTUC compared with adjacent normal tissues. The regulatory role of miR-30a-5p in UTUC progression was further validated using a UTUC cell line (BFTC-909). The functional assays including cell growth, migration, and the underlying mechanisms between miR-30a-5p and the EMT were investigated. 

## 2. Results

### 2.1. Profiling the miRNA Expression Signature of UTUC by NGS and qPCR

**Figure 1:**
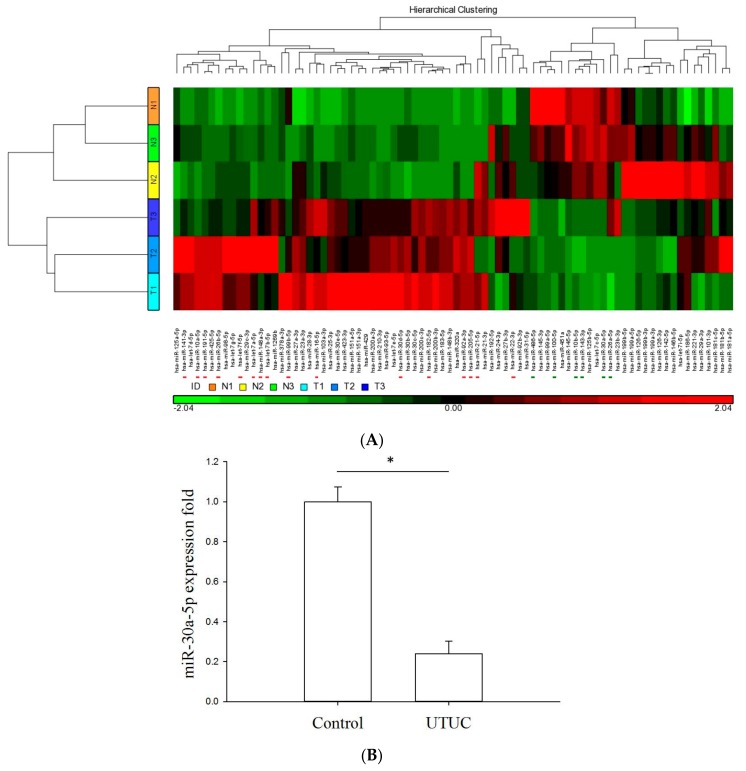
Expression profiles of miRNAs in human UTUC tumor and non-tumor samples. Three tumor (T1–T3; light blue to navy blue) and three adjacent normal tissues (N1–N3; orange-yellow-green) were subjected to RNA extraction and NGS-miRNA sequencing through the Illumina MiSeq platform. (**A**) Heat-map clustering analysis was conducted to examine the overall miRNA expression profiles among samples. A line scatterplot was used to present miRNA expression profiles; (**B**) Downregulation of miR-30a-5p in UTUC tumor tissues (*n* = 22) compared with adjacent normal controls (*n* = 14) by RT-qPCR analysis. The asterisk denotes *p* < 0.001 using an unpaired *t*-test.

In this study, we used NGS for genome-wide miRNA profiling, followed by specific validation with quantitative polymerase chain reaction (qPCR). We first had three UTUC samples and three adjacent normal tissues sequenced with an Illumina platform. As a result, approximately five million sequence reads were generated for each library. The generated sequence reads were analyzed with the miRSeq toolkit [20]. According to the miRSeq analysis result, the miRNA NGS data were high-quality with clean reads (the reads with the 3′ adaptor were identified and trimmed). All libraries demonstrated a dominance in size 22-nt, consistent with known human miRNAs. In addition, most of the sequence reads belonged to miRNAs, reflecting the good performance of sample preparation. Subsequent clustering analysis was performed to examine the overall miRNA expression profile. As shown in Figure 1A, the clinical samples were clearly and exactly clustered, distinguishing between normal versus tumor. Figure 1A also shows that many miRNAs were differentially expressed between normal and tumor tissue samples. In addition, among the differentially-expressed miRNAs, miR-30a-5p was significantly downregulated in UTUC samples (*p* < 0.05). Moreover, the qPCR validation showed that the miR-30a-5p expression level was higher in the control samples (Figure 1B). These data implied that the putative function of miR-30a-5p may act as a tumor repressor.

### 2.2. Transcriptome Profiles in UTUC Tissues on Illumina HT12 Microarray Chips

**Figure 2:**
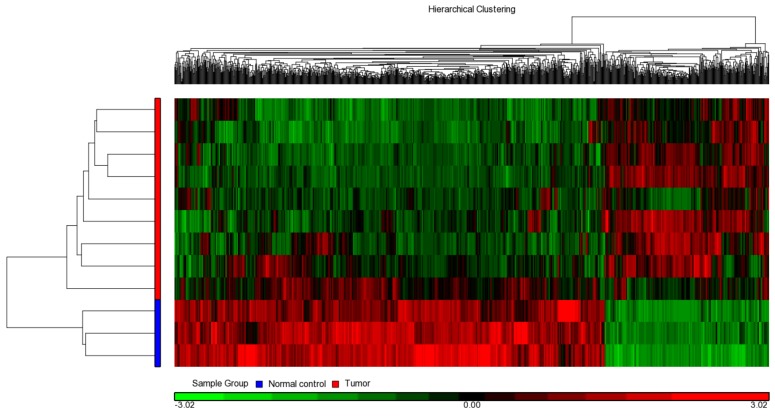
Comparative transcriptome analysis in human UTUC tumor and non-tumor samples. The total RNA isolated from human UTUC tumors (*n* = 9) and adjacent normal tissues (*n* = 3) was subjected to transcriptome analysis on Illumina HT12 microarray chips. The expression data was used for heat-map clustering analysis.

The miRNAs performed downregulation abilities by repressing their target protein-coding genes. To investigate the possible regulation roles of miRNAs in UTUC, we first derived a comprehensive understanding of gene expression profiles. We had three adjacent normal as well as nine UTUC tissues examined with Illumina HT12 microarray chips. After RNA sample preparation, hybridization, and scanning, we identified 1135 genes differentially expressed between normal and tumor tissues (*p* < 0.05 and expression ratio > 2). Among them, 314 and 821 genes were upregulated and downregulated in tumors, respectively. These differentially-expressed genes were used for heat map clustering analysis. As a result, normal and tumor samples were clearly and exactly clustered (Figure 2).

### 2.3. Pathway Analysis on the Involvement of miRNAs in the Tumorigenesis of UTUC

**Figure 3:**
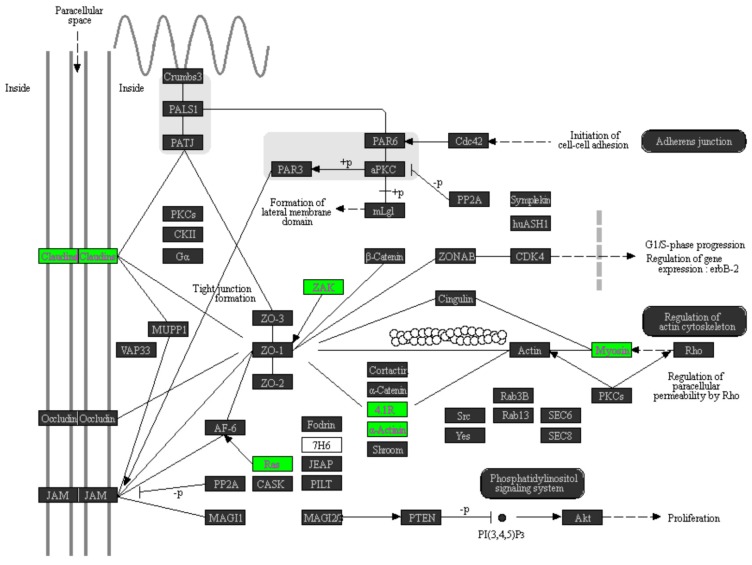
KEGG pathway enrichment analysis on the tight junction pathway. The differentially-expressed genes in human UTUC tumor tissues compared to adjacent normal tissues were subjected to KEGG pathway enrichment analysis using Partek Genomics software. Note that a total of six genes (highlighted in green boxes) in the tight junction pathway, including claudin-5, were significantly downregulated in UTUC tumor tissues.

A previous study investigated whether different miRNAs simultaneously acted on the same pathway [21]. In this study, we also investigated whether the simultaneously upregulated or simultaneously downregulated miRNAs contributed to the pathogenesis of UTUC. For this purpose, we collected two sets of miRNAs. The first set included six downregulated miRNAs (highlighted with the green bar in Figure 1A) in tumors and the second one comprised 16 upregulated miRNAs (highlighted with the red bar in Figure 1A) in tumors. Among the 314 upregulated genes detected by microarray, 38 are the target genes of the six downregulated miRNAs according to TargetScan 6.2 annotation. Among the 821 downregulated genes, 259 are the target genes of the 16 upregulated miRNAs according to TargetScan 6.2 annotation. We next performed pathway enrichment analysis on the 38 (Table S1) and 259 gene (Table S2) as per a previous study [22]. As listed in Table 1, Kyoto Encyclopedia of Genes and Genomes (KEGG) pathway enrichment analysis indicated that 19 biological and signal pathways were affected in UTUC tissues, while the top three pathways included MAPK (Figure S1), PI3K/Akt (Figure S2), and tight junction (TJ) pathways (Figure 3). It has been determined that TJ is an essential structure that maintains the contacts between epithelial cells and determines the epithelial cell polarity, and the expression of TJ structural proteins including claudins was markedly decreased in EMT during tumor metastasis [23,24,25]. Moreover, the altered expression of claudin proteins has been found to closely associate with the tumor stage and survival of UTUC patients [26]. We were particularly interested in the regulatory roles of miR-30a-5p in the proliferation, migration, EMT, and TJ protein expression of UTUC cells.

### 2.4. miR-30a-5p Overexpression Reduced Proliferation and Migration of BFTC-909 Cells

**Figure 4:**
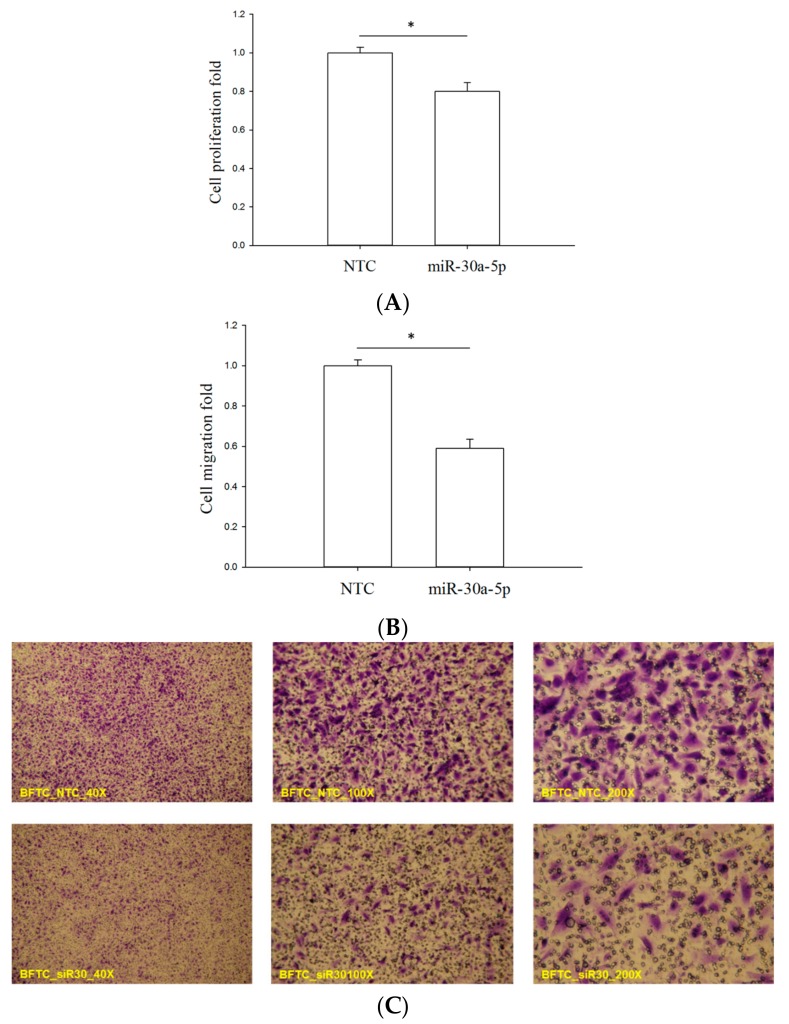
The suppressive effect of miR-30a-5p on the proliferation and migration of UTUC cells. BFTC-909 cells were transfected with miR-30a-5p mimetic agent, followed by WST-1 and trans-well assays. NTC denotes non-transfection controls with scrambled mimetic treatment. (**A**) After mimetic transfection for 48 h, the WST-1 assay was used to evaluate cell numbers. The WST-1 value is directly proportional to cell number; (**B**) After mimetic transfection for 72 h, trans-well migration assay was performed to evaluate the cell migration ability; (**C**) The crystal violet-stained cells are those penetrating the trans-well membrane. All data are shown as means ± SEM from three independent experiments. * indicates that *p* < 0.001 between the indicated groups.

To investigate the possible effect of miR-30a-5p on proliferation and migration, mimics of miR-30a-5p were transfected into BFTC-909 cells, which is the well-known in vitro model of UTUC [27]. Water-soluble tetrazolium (WST-1) assay [28] and trans-well assay [29] were used to measure cell number and cell migration ability, respectively. The value of the former is directly proportional to cell number, providing a quantitative indicator of cell number. For the latter assay, the number of cells penetrating through the membrane of the trans-well provides a quantitative indicator of cell migration ability. The assay results clearly showed that miR-30a-5p overexpression significantly reduced cell number (Figure 4A, *p* < 0.05) and migration (Figure 4B,C, *p* < 0.05). These data demonstrated the suppressive effect of miR-30a-5p on cancer cell growth and migration, and strongly suggested that it could serve as a tumor repressor in UTUC cells.

### 2.5. miR-30a-5p Overexpression Inhibits Epithelial-to-Mesenchymal Transition (EMT) in BFTC-909 Cells

**Figure 5:**
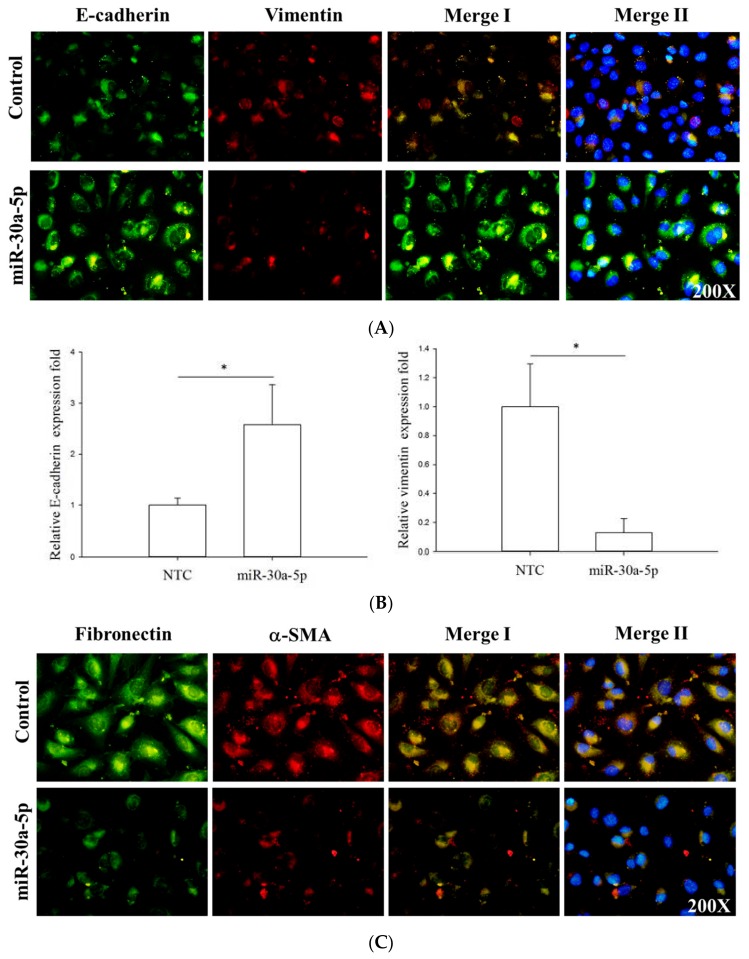

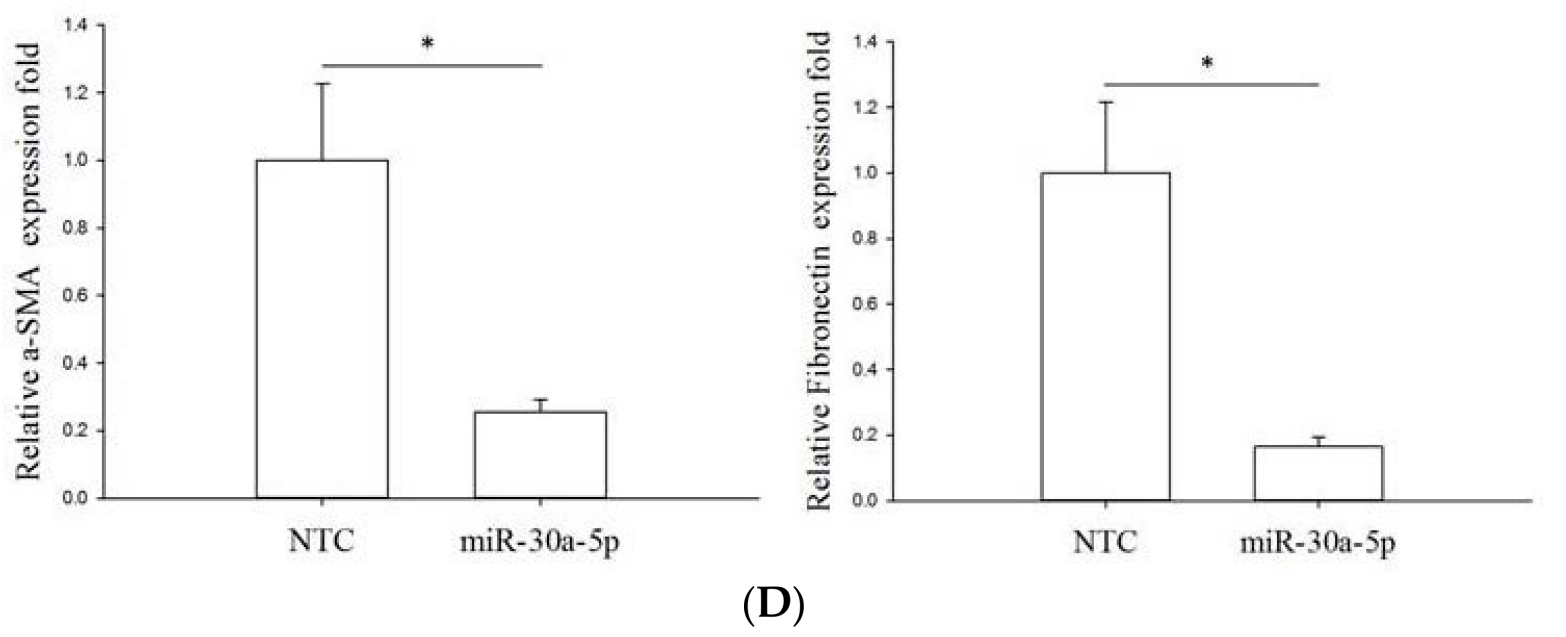
miR-30a-5p overexpression inhibited epithelial-to-mesenchymal transition in UTUC cells. The BFTC-909 cells transfected with miR-30a-5p were subjected to immunofluorescent staining for cellular distributions of (**A**) E-cadherin (red), vimentin (green), and nuclei (blue); (**C**) Alternatively, α-SMA (red), fibronectin (green), and nuclei (blue) were immunofluorescently visualized. The fluorescence intensities of E-cadherin and vimentin (**B**), as well as EMT markers, α-SMA, and fibronectin (**D**), were quantified by counting 5–10 different fields per sample. NTC denotes non-transfection controls with scrambled mimetic treatment. Data are expressed as means ± SEM (*n* = 3). * indicates that *p* < 0.05 between the indicated groups. Original magnification: 200×.

**Figure 6:**
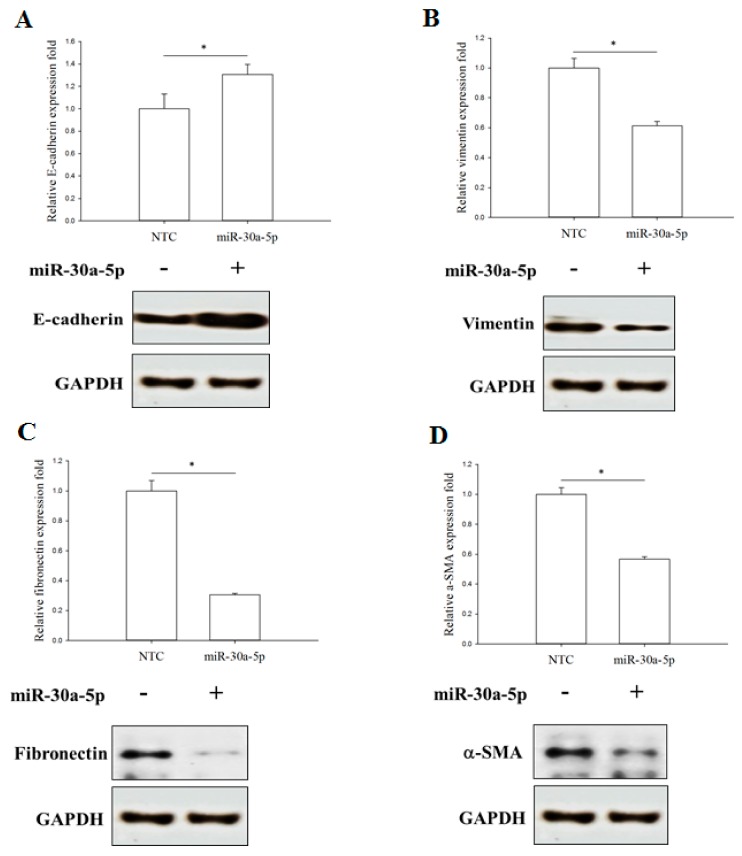
miR-30a-5p overexpression inhibits epithelial-to-mesenchymal transition marker expression in UTUC cells. Western blot analysis of expression levels of epithelial marker E-cadherin (**A**), and mesenchymal markers, including vimentin (**B**), fibronectin (**C**), and α-SMA (**D**) in cultured BFTC-909 cells transfected with miR-30a-5p or miR-NC. GAPDH was used as the loading control. Data are expressed as means ± SEM (*n* = 3). * indicates that *p* < 0.05 between the indicated groups.

To investigate whether miR-30a-5p could affect the EMT process in UTUC, we transfected the miR-30a-5p mimetics into BFTC-909 cells and the expression levels of EMT markers, including E-cadherin, vimentin, α-smooth muscle actin (α-SMA), and fibronectin, were examined. The immunofluorescence data showed that overexpression of miR-30a-5p significantly restored E-cadherin and inhibited the expression of α-SMA, fibronectin, and vimentin (Figure 5). These data were further confirmed by Western blot analysis. Similarly, miR-30a-5p overexpression significantly enhanced the expression of E-cadherin and reduced α-SMA, fibronectin, and vimentin protein expression (Figure 6). Taken together, these data indicated that miR-30a-5p might inhibit the EMT process in UTUC cells.

### 2.6. miR-30a-5p Overexpression Increased TJ Protein Claudin-5 Expression in BFTC-909 Cells

**Figure 7:**
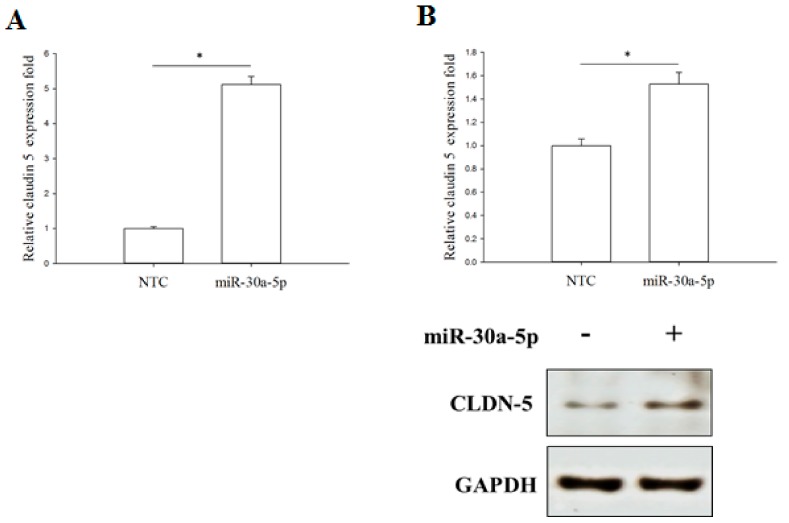
Claudin-5 expression is enhanced by the miR-30a-5p. The qPCR (**A**) and Western blot (**B**) analysis of *CLDN-5* expression levels in cultured BFTC-909 cells transfected with miR-30a-5p or miR-NC. GAPDH was used as the loading control. Data are expressed as means ± SEM (*n* = 3). * indicates that *p* < 0.05 between the indicated groups.

According to the pathway enriched analysis results illustrated in Figure 3, claudin-5 (*CLDN-5*) expression was significantly downregulated in UTUC tissue samples compared with normal renal pelvis mucosa. Thus, we further investigated the role of miR-30a-5p in the regulation of *CLDN-5* expression. RT-qPCR and Western blotting analysis demonstrated that miR-30a-5p overexpression significantly increased the *CLDN-5* mRNA and protein expression in BFTC-909 cells (Figure 7). These results demonstrated that the reduction of *CLDN-5* expression in UTUC might be restored by miR-30a-5p delivery. In summary, these results indicated that miR-30a-5p might inhibit the EMT process by increasing *CLDN-5* expression of the TJ pathway during UTUC tumorigenesis. Thus, miR-30a-5p might be a good therapeutic target for UTUC treatment.

## 3. Discussion

Aberration expressions of miRNAs were noted in sera and tissues in many human cancers a decade ago. It is increasingly recognized as a novel regulatory mechanism in cancer development [5,6,7,8,9]. Certain miRNAs have been found to be downregulated in various types of solid tumors and involved in the tumorigenic processes. Thus, a better understanding of the gene regulatory network orchestrated by these miRNAs may help exploit the potential application of miRNA biomarkers in cancer diagnosis as well as targeting strategy in cancer treatment.

In light of the fact that the roles of miRNAs in UTUC are not fully elucidated, this study characterized and compared the miRNA expression profiles between human UTUC and adjacent normal tissues, and analyzed the relationship between the downregulated miRNAs and the dysregulated pathways involved in tumorigenic processes. We identified the reduction of miR-30a-5p is one of the miRNA signatures in UTUC tissues. In fact, previous studies have reported that miR-30a plays a tumor suppressive role and is downregulated in liver, prostate, and breast cancers, which is well correlated with unfavorable clinical outcomes [30,31,32]. Our results demonstrated that miR-30a-5p expression was significantly lower in UTUC tissues compared to adjacent non-cancerous tissues, implicating its involvement in the proliferation and metastasis of UTUC cells. Similar to our findings, the suppressive role of miR-30a in the bladder UC has more recently been demonstrated in a study by Zhang et al., in which they noted not only the reduction of miR-30a, but also its close association with shorter overall survival and disease-free survival of patients [33]. To support this notion, the KEGG pathway analysis data in this study also showed the aberration of Notch signaling in the human UTUC tissue samples (Figure S3). In agreement with our in vitro findings, miR-30a has been found to suppress the cell proliferation, migration, and invasiveness of many cancer cells [32,33,34,35], including bladder cancer [33]. Moreover, our data of pathway enrichment analysis also highlights the involvement of MAPK and PI3K/Akt pathways in the tumorigenesis of UTUC (Table 1; Figures S1 and S2). The MAPK signaling activation in the carcinogenesis of invasive bladder transition cell carcinomas has been addressed by a meta-analysis study [36], whereas the PI3K/Akt pathway is implicated as the target of miR-30a to overcome EGFR inhibitor resistance in lung cancer [37].

In addition to the modulatory effects on tumor cell behaviors in vitro, the present study data also demonstrated that miR-30a-5p genetic delivery in UTUC BFTC-909 cells could restore the expression of the epithelial marker E-cadherin, and concomitantly suppress the expression of mesenchymal markers, including vimentin, fibronectin, and α-SMA proteins. It is well known that EMT is a prerequisite process that contributes to the metastatic ability and therapy resistance in different types of cancers [18]. Taken with earlier studies [16,17], this study confirmed the inhibitory effect of miR-30a-5p on the EMT of UTUC cells. 

**Figure 8:**
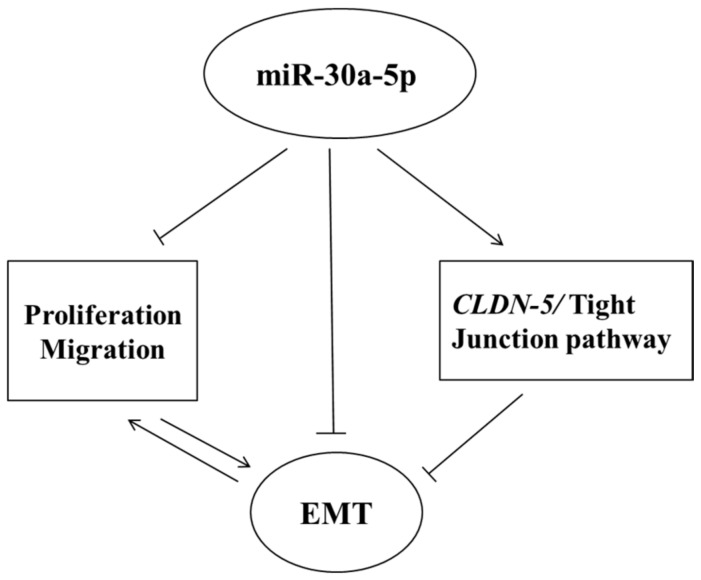
Proposed effects of miR-30a-5p on UTUC. The miR-30a-5p-enhanced *CLDN-5* expression may trigger tight junction pathway signal transduction. Moreover, miR-30a-5p suppresses the EMT process via increasing E-cadherin and suppressing vimentin, fibronectin, and α-SMA expression, thereby inhibiting the migration activity of UTUC cells.

As claudins are major constituents of TJ structure proteins that maintain intercellular adhesion and determine epithelial cell polarity, reduction, or loss of their constitutive expression has been found to correlate with high tumor grade and disease recurrence in many cancers [23,25]. Although previous immunohistochemistry studies have shown aberrant expression of claudin-1, -3, -4, and -7 proteins in human tissues of UTUC [26] and bladder cancers [38], the expression of claudin-5 and its regulatory role in TJ homeostasis during UTUC tumorigenesis still remains elusive. Other lines of evidence indicate that claudin-5 expression is prominently decreased in human lung squamous cell carcinoma, whereas exogenous transfection of claudin-5 genes dramatically suppress the proliferation of lung cancer cells, possibly via inhibiting Akt phosphorylation [39]. With regard to the regulatory role of miRNAs in the functioning of TJ proteins and EMT homeostasis, miR-30a overexpression has recently been reported to reduce filopodia formation and the metastastic capability of breast tumor cells, and concomitantly increase claudin expression therein, possibly via targeting Slug signaling [18]. More intriguingly, the present study provides the first in vitro evidence showing that miR-30a-5p overexpression significantly increased claudin-5 expression in UTUC BFTC-909 cells, raising the possibility that miR-30a-5p is very likely to reverse EMT through disrupting the functioning of regulators in the TJ pathway. The modulatory effect of miR-30a-5p in UTUC cells is depicted in Figure 8. Further work is warranted to evaluate the downstream targets regulating the TJ pathway and to develop therapeutic strategies targeting miR-30a-5p in vivo.

## 4. Materials and Methods

### 4.1. Clinical Specimens

We collected tumor tissues and adjacent normal tissues from 22 patients who were diagnosed with UTUC of the renal pelvis (Table S3) and had undergone nephroureterectomy in Kaohsiung Chang Gung Memorial Hospital during 2015. The adjacent normal samples were collected from the normal renal pelvis mucosa (non-cancer areas) grossly. Part of the tissue was sent for exclusion of carcinoma in situ. None of the patients in this study underwent radiotherapy or chemotherapy before surgery. Informed consent was obtained from all patients according to local ethical regulations, and the protocol was approved by the Institutional Review Board of Chang Gung Memorial Hospital (IRB No. 101-4722B, approval date: 23/December/2013) and complied with the Declaration of Helsinki. Histological examination and clinical diagnosis of the tumors and normal adjacent tissues from the renal pelvis in patients were performed. Fresh tissues were immediately immersed in RNA later (Qiagen; Hilden, Germany) after surgical resection and stored at 4 °C overnight to allow thorough penetration of the tissues, which were thereafter stored at −80 °C.

### 4.2. RNA Isolation

Total RNA was extracted by Trizol^®^ Reagent (Invitrogen, Waltham, MA, USA) according to the instruction manual. The quality of isolated RNA was analyzed by using an RNA 6000 Lab-chip kit (Agilent Technologies, Santa Clara, CA, USA) on a Bioanalyzer 2100 (Agilent Technology, Santa Clara, CA, USA) and quantified at OD 260 nm by using an ND-1000 spectrophotometer (Nanodrop Technology, City, State, USA).

### 4.3. Library Preparation and Sequencing

The small RNA library construction and deep sequencing was carried out at a Biotechnology Company (Welgene, Taipei, Taiwan). Samples were prepared using an Illumina sample preparation kit according to the TruSeq Small RNA Sample Preparation Guide. The 5′ and 3′ adaptors were ligated to total RNA followed by reverse transcription (RT) and real-time polymerase chain reaction (PCR) amplification. The enriched cDNA constructs were size-fractionated and purified on a 6% polyacrylamide gel electrophoresis and the bands containing the 18–40 nucleotide RNA fragments (140–155 nucleotides in length with both adapters). Libraries were sequenced on an Illumina instrument (75 cycle single read).

### 4.4. Pathway Enrichment Analysis

After the significantly-expressed genes were identified, pathway enrichment analysis was applied to select for target genes for subsequent further validation or functional assays. Under the assumption that genes with similar expression have tendencies to be functionally associated, pathway enrichment analysis was performed in terms of pathways rather than in terms of single genes. Commercial analysis software (Partek Genomics Suite, version 6.6, St. Louis, MO, USA) was used to map the target genes to KEGG pathways, calculate *p*-values, and identify the significantly-enriched pathways.

### 4.5. Reverse Transcription (RT) and Real-Time PCR

To prepare a cDNA pool from each RNA sample, total RNA (10 ng) was reverse-transcribed using a TaqMan MicroRNA reverse transcription kit (ABI, catalog no. 4366596) according to the manufacturer’s instructions. Each cDNA pool was stored at −20 °C until further real-time PCR analysis. A TaqMan probe assay kit, containing RT primer, forward and universal reverse primers, MGB probe was purchased from ABI for real-time PCR assays. Real-time PCR reactions were performed on AriaMx Real-Time PCR system using Brilliant III QPCR Master Mixes with low ROX (Agilent, catalog No. 600890, Santa Clara, CA, USA). Briefly, 20-μL reactions contained 10 μL of 2X Brilliant III QPCR Master Mixes, 1 μL of TaqMan microRNA assay primers, and 2 μL cDNA samples. Each sample was run in triplicate. The RT-PCR program ran at 95 °C for 3 min, 50 cycles of 95 °C for 5 s, and 60 °C for 10 s. At the end of each RT-PCR run, the data were automatically analyzed by the system and an amplification plot was generated for each cDNA sample. From each of these plots, the AriaMx Data analysis software automatically calculated the *C*q value (quantification cycle, the cycle at which fluorescence is determined to be statistically significant above the background signal contributed by the fluorescently-labeled oligonucleotides within the PCR reaction), which implies the beginning of exponential amplification. The fold expression or repression of the target gene relative to the internal control gene U6 snRNA and Glyceraldehyde-3-Phosphate Dehydrogenase (GAPDH) used for *CLDN-5* study in each sample was then calculated by the formula: 2^−∆∆*C*q^, where ∆*C*q = *C*q_target gene_ − *C*q_internal control_ and ∆∆*C*q = ∆*C*q_test sample_ − ∆*C*q_control sample_

### 4.6. Cell Culture and miRNA Treatment

A UTUC cell line, clone BFTC909, was cultured as described previously [27]. To observe the biomodulatory effect of miR-30a-5p on UTUC cell behaviors, the cells were stably transfected with miR-30a-5p mimetic agent following the manufacturer’s instructions and the guidelines for miRNA mimic and miRNA inhibitor experiments (Qiagen, Hilden, Germany).

### 4.7. Cell Proliferation Assay

Cell proliferation assay was performed by using a WST-1 assay kit (TaKaRa catalog No. MK400, Mountain View, CA, USA). Cells were seeded on microtiter plates (5 × 10^4^ cells/well) in a final volume of 100 μL culture medium per well in a humidified atmosphere (e.g., 37 °C, 5% CO_2_). After 48 h of incubation, 10 μL/well Premix WST-1 was added and the cells were incubated for another 4 h in a humidified atmosphere (e.g., 37 °C, 5% CO_2_). Afterward, the absorbance of the formazan end-product in each well and background blank control at 440 nm, as well as the reference wavelength at 600 nm, were measured using a microtiter plate (ELISA) reader (Sunrise, Tecan, Männedorf, Switzerland).

### 4.8. Trans-Well Cell Migration Assay

The cell migration assay used Trans-well chambers (Millicell, PIEP12R48, Billerica, MA, USA). DMEM medium containing 10% serum was used as an attractant in the lower chamber. To the upper chamber of the insert were placed 0.3 × 10^6^ transfection cells in DMEM medium without serum, allowed to migrate toward the underside of the insert filter at 37 °C for 24 h. Cells on the lower side of the insert filter were fixed by ice methanol and stained with crystal violet solution. The numbers of cells on the underside of the filter from six randomly-selected microscopic fields were counted.

### 4.9. Western Blot Analysis

Cellular protein lysates were prepared by homogenization with 1× RIPA lysis buffer (Cell Signaling Technology, Billerica, MA, USA). Protein concentrations were measured using a protein assay dye (Bio-Rad Laboratories, Hercules, CA, USA). SDS-PAGE and immunoblotting analysis were performed as described previously [40]. The detecting antibodies were raised against E-cadherin (ab76055, Abcam, UK), vimentin (2707-1 epitomics), α-SMA (ab5694, Abcam, Cambridge, UK), fibronectin (ab2413, abcam), claudin 5 (ab15106, Abcam, Cambridge, UK), and GAPDH (GTX627408, GeneTex, Irvine, CA USA).

### 4.10. Immunofluorescent Staining

BFTC909 cells were cultured in six-well glass slide chambers for 24 h and further transfected with miR-30a-5p mimetics for 48 h. The cells were then fixed with 4% paraformaldehyde, permeabilized with 0.25% Triton X-100, and blocked with 3% BSA for 30 min at room temperature. The fixed cells were then incubated with the primary antibodies against E-cadherin, vimentin, fibronectin, and α-SMA at 4 °C overnight, followed by visualization with Alexa Fluor 488 (green) or Alexa Fluor 595 (red)-conjugated secondary antibodies at room temperature for 1 h. Nuclei were counterstained with DAPI. The stained cells were mounted with a fluorescent mounting medium (Dako Cytomation) and observed under fluorescence microscopy (Olympus, Tokyo, Japan). The exposure gains and rates were consistent between samples. Fluorescent intensities were quantified on independent color channels by using Image J software (NIH, stapleton staten island, NY, USA).

## 5. Conclusions

In conclusion, this study demonstrated that the downregulation of miR-30a-5p is characteristic of UTUC and suppresses the proliferation and migration of UTUC cells. Moreover, miR-30a-5p gene delivery inhibits EMT and upregulates the expression of tight junction protein claudin-5. These findings suggest that miR-30a may be a candidate miRNA biomarker applicable for the prognostic assessment and/or targeting therapy for UTUC in the future.

## Figures and Tables

**Table 1:** Result of pathway enrichment analysis on the 259 downregulated genes. We identified 19 significant pathways, the *p*-values, pathway names, and KEGG pathway IDs of which are provided.

Pathway Name	*p*-Value	Pathway ID
MAPK signaling pathway	0.00274259	kegg_pathway_261
PI3K-Akt signaling pathway	0.00308062	kegg_pathway_262
Tight junction	0.00451891	kegg_pathway_257
Protein digestion and absorption	0.00458404	kegg_pathway_279
Notch signaling pathway	0.00534759	kegg_pathway_55
Cytokine-cytokine receptor interaction	0.00924043	kegg_pathway_79
Endocytosis	0.0151682	kegg_pathway_232
Focal adhesion	0.0158629	kegg_pathway_188
Regulation of actin cytoskeleton	0.0164765	kegg_pathway_139
Morphine addiction	0.0191389	kegg_pathway_101
Calcium signaling pathway	0.022008	kegg_pathway_237
Amoebiasis	0.0234208	kegg_pathway_214
Amino sugar and nucleotide sugar metabolism	0.0254128	kegg_pathway_203
GABAergic synapse	0.0258019	kegg_pathway_235
Ras signaling pathway	0.0265302	kegg_pathway_265
Endocrine and other factor-regulated calcium reabsorption	0.0266089	kegg_pathway_174
HTLV-I infection	0.0280212	kegg_pathway_190
ECM-receptor interaction	0.0314539	kegg_pathway_242
Jak-STAT signaling pathway	0.0400457	kegg_pathway_40

## Supplementary Materials

Supplementary materials can be found at www.mdpi.com/1422-0067/18/8/1826/s1.
