# Supplementary material for: MiR-30a-5p Inhibits Epithelial-to-Mesenchymal Transition and Upregulates Expression of Tight Junction Protein Claudin-5 in Human Upper Tract Urothelial Carcinoma Cells
Source: Int J Mol Sci. 2017 Aug 22;18(8):1826. doi: 10.3390/ijms18081826 (PMC5578210; doi:10.3390/ijms18081826)

Supplementary Figures

Figure S1. KEGG pathway enrichment analysis on the MAPK signaling pathway. The microarray-detected transcriptome profiles of human upper tract urothelial carcinoma and adjacent normal tissues were compared and mapped by using a Partek Genomics software for pathway analysis. The up-regulated genes are highlighted in green boxes.

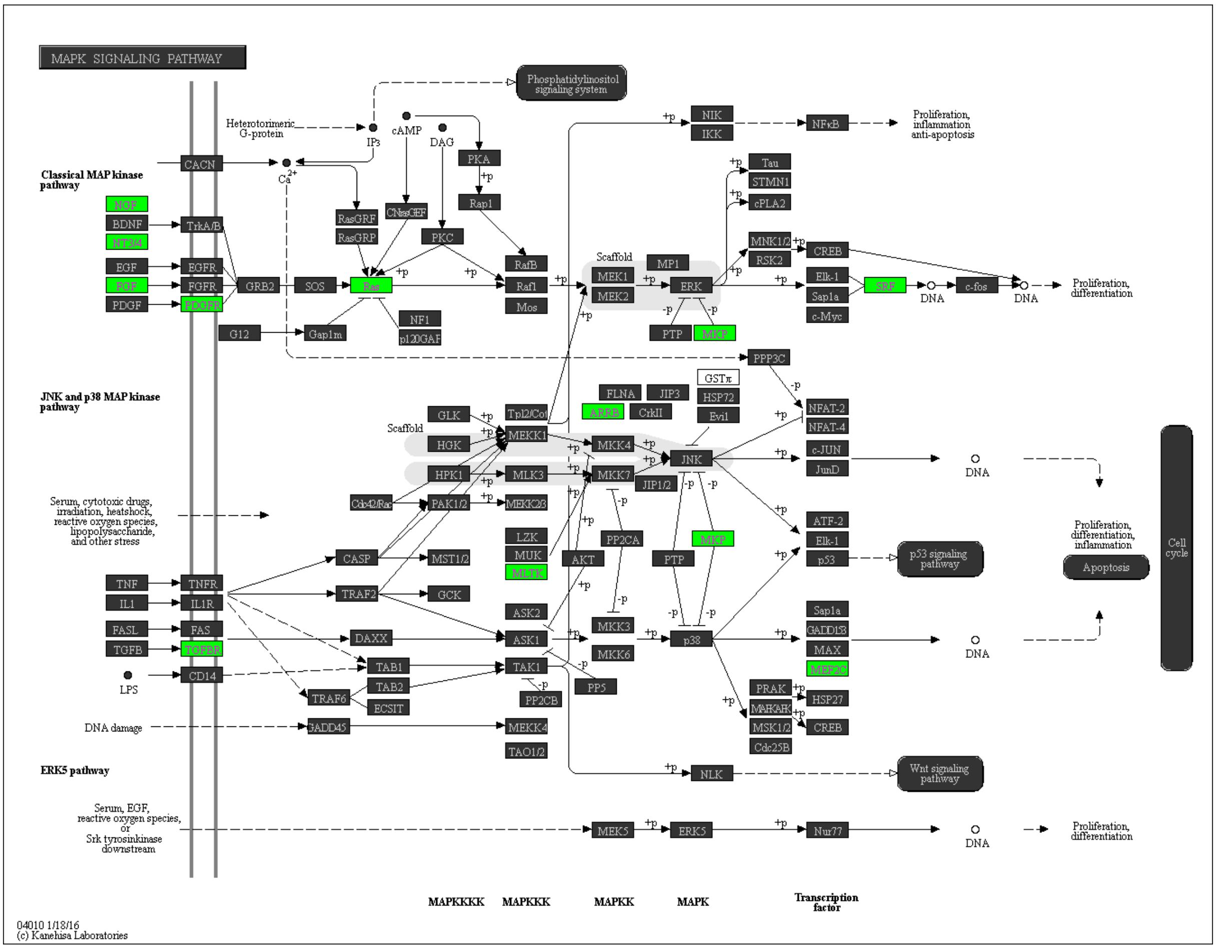

Figure S2. KEGG pathway enrichment analysis on the PI3K-AKT signaling pathway. The microarray-detected transcriptome profiles of human upper tract urothelial carcinoma and adjacent normal tissues were compared and mapped by using a Partek Genomics software for pathway analysis. The up-regulated genes are highlighted in green boxes.

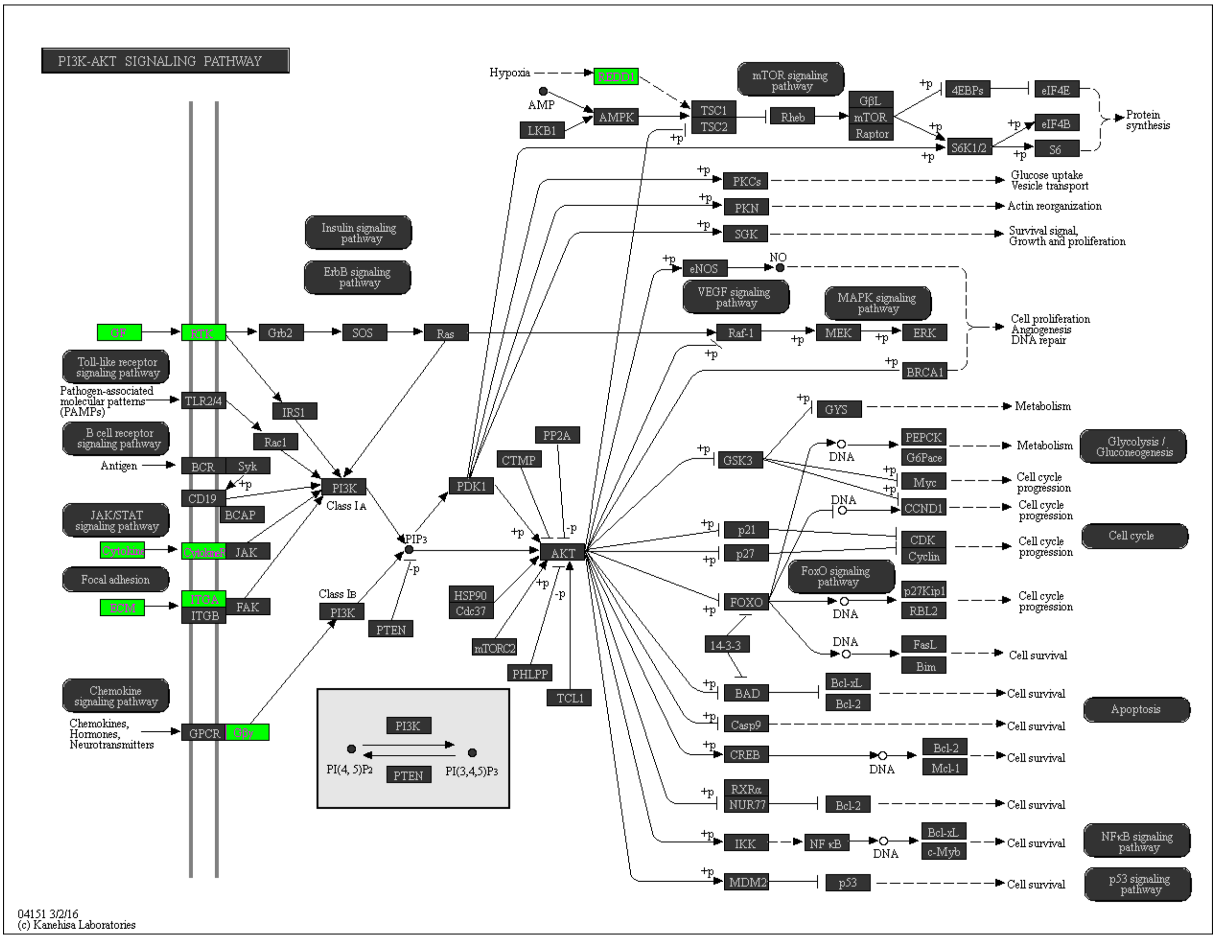

Figure S3. KEGG pathway enrichment analysis on the NOTCH signaling pathway. The microarray-detected transcriptome profiles of human upper tract urothelial carcinoma and adjacent normal tissues were compared and mapped by using a Partek Genomics software for pathway analysis. The up-regulated genes are highlighted in green boxes.

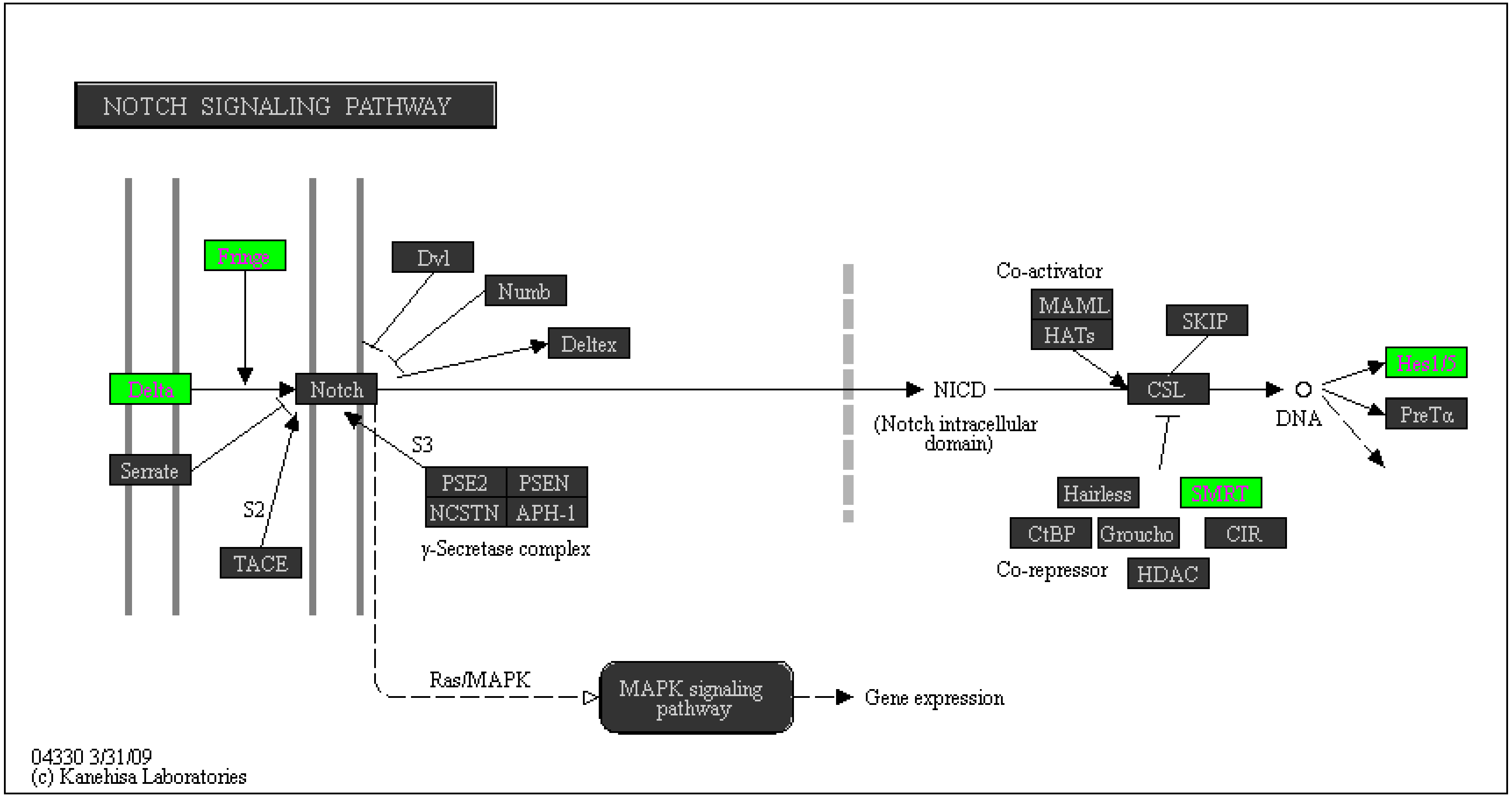

Supplement: Supplementary file 1 [file ijms-18-01826-s001.zip › 1. ijms-213753-suppl-Figures.pdf]
