# Supplementary material for: MiR-30a-5p Inhibits Epithelial-to-Mesenchymal Transition and Upregulates Expression of Tight Junction Protein Claudin-5 in Human Upper Tract Urothelial Carcinoma Cells
Source: Int J Mol Sci. 2017 Aug 22;18(8):1826. doi: 10.3390/ijms18081826 (PMC5578210; doi:10.3390/ijms18081826)
Supplement: Supplementary file 1 [file ijms-18-01826-s001.zip › Supplementary Tables .pdf]

Supplementary Tables

Table S1. Result of pathway enrichment analysis on the 38 genes differentially expressed in the 6 down-regulated miRNAs.

Gene symbol of 38 up-regulated target genes				
ARID4B	CASK	CCNE2	CDC7	CDCA7
CHD7	CKS2	E2F2	EZH2	FAM60A
FBXO28	GCLC	GPR180	GRHL2	HMGB3
HOOK1	KCNK1	KLF5	KPNA2	LPAR2
LRRC8D	MAP7	MTDH	MTF2	PGK1
PTPRK	RAB3IP	RCN2	SIX4	SLC38A1
SNRPD1	SOX4	SPCS3	TAOK1	TIA1
TP63	UCK2	XPO1		

Table S2. Result of pathway enrichment analysis on the 259 genes differentially expressed in the 16 up-regulated miRNAs.

Gene symbol of 259 down-regulated target genes				
ACACB	ACTN1	ACVRL1	ADAMTS1	ADAMTSL3
ADCY9	ADRB2	AHNAK	AKAP12	ALAD
ANGPTL2	ANTXR2	APOLD1	ARHGAP20	ARHGEF10
ARL2	ARRB1	ATOH8	ATP1A2	ATP8B2
ATP8B4	AXL	BIN1	CALD1	CAPN5
CBX6	CBX7	CCDC28A	CCDC80	CCDC92
CCPG1	CDC42EP2	CDH11	CDH5	CGNL1
CILP	CLDN11	CMTM7	CNN1	COL14A1
COL15A1	COL4A2	COL5A1	CORO1C	CRY2
CSF1R	CTGF	CX3CL1	CXCL12	CYR61
CYYR1	DAB2	DCLK1	DCUN1D3	DDIT4
DIXDC1	DKK3	DLL1	DPYSL2	DPYSL3
DUSP1	EBF1	EBF3	EDN1	EDNRA
EML1	EMP1	EMX2	EPB41L2	EPDR1
ESR1	F13A1	FAM107A	FAM43A	FAM46A
FERMT2	FGF9	FLRT2	FOSB	FOXC1
FOXD1	FOXF1	FRZB	FSTL1	FZD4
G0S2	GABARAPL1	GABBR2	GALNTL2	GAS1
GFPT2	GHR	GLIPR2	GMDS	GNE
GNG7	GPD1	GPR116	GPR124	GPR162
HABP4	HEG1	HES1	HLF	HOXC8

HTR2A	IL6	INHBB	ISLR	ITGA9
ITPRIP	ITSN1	JUNB	KCTD12	KIAA1210
KLF2	KLF4	KLF6	KLF9	LAMA4
LEPR	LGALS1	LIMS2	LIPE	LMOD1
LOX	LRP1	LRRC17	MAF	MAP1A
MAP1B	MATN2	MEF2C	MFGE8	MFNG
MGLL	MPRIP	MRAS	MSRB3	MYADM
MYH10	MYH11	MYLK	NCALD	NCOR2
NFIA	NFIX	NGF	NTF3	NTM
OAF	OGN	PALLD	PAPPA	PARM1
PCDH18	PCOLCE2	PDE5A	PDGFRA	PDGFRB
PDK4	PDPN	PDZD4	PHLDB1	PI16
PID1	PIM1	PKDCC	PLCXD3	PLEKHO2
PLSCR4	PMEPA1	PPAP2B	PRELP	PRICKLE1
PRICKLE2	PROM1	PRRX1	PTPRN2	RAB11FIP2
RAB23	RAB9B	RASD1	RASL12	RASSF2
RBPMS2	RECK	REEP1	RFTN1	RFTN2
RGL1	RGS2	RGS4	RHOB	RHOJ
RNF150	RRAD	RSPO3	SCARA3	SCARA5
SCN11A	SCN4B	SCRG1	SDC2	SERPINE1
SH3D19	SH3KBP1	SH3PXD2A	SHANK3	SIDT2
SIK1	SLC24A3	SLC2A3	SLC7A2	SLC9A9
SOCS3	SORBS1	SORBS2	SOX17	SPRY1
SRF	SRL	ST6GALNAC6	STX2	SULF2
SVEP1	SYDE1	SYNPO2	SYT11	SYTL2
TACC1	TAGLN	TCF21	TCF4	TGFBI
TGFBR2	THRSP	TIMP2	TIMP3	TMOD1
TNFSF13B	TNS1	TP53INP2	TPPP3	TSC22D3
TSHZ3	TSKU	TSPAN33	TSPAN4	TSPAN9
VASN	ZAK	ZBTB20	ZBTB47	ZCCHC24
ZDHHC14	ZEB2	ZFP36	ZHX3	

Table S3. Clinical pathological characteristics of 22 patients with upper tract urothelial carcinoma.

Characteristic Variables	Number of patients (*n*)
**Gender**	
Male	9
Female	13

**Age**	
<60	4
≥60	18
**Tumor status (TMN)**	
T1-2	12
T3-4	10
N0	15
N1	7
M0	9
M1	13
**Grade**	
High	17
Low	5
**Tumor necrosis**	
Yes	10
no	12
